# First Isolation of *West Nile virus* from a Patient with Encephalitis in the United States

**DOI:** 10.3201/eid0812.020532

**Published:** 2002-12

**Authors:** Cinnia Huang, Brett Slater, Robert Rudd, Nandakishore Parchuri, Rene Hull, Michelle Dupuis, Alexander Hindenburg

**Affiliations:** * Wadsworth Center, New York State Department of Health, Albany, New York, USA; † Winthrop University Hospital, Mineola, New York, USA

**Keywords:** West Nile virus isolation, RT-PCR, lymphoma, rituximab, chemotherapy, immunodeficiency, CSF

## Abstract

*West Nile virus* (WNV) was isolated from a patient who developed encephalitis while undergoing treatment with CHOP (cyclophosphamide, hydroxydoxorubicin, vincristine [Oncovin], predisone) and rituximab for a non-Hodgkin B-cell lymphoma. Both standard reverse transcription–polymerase chain reaction (RT-PCR) and Taqman RT-PCR established the diagnosis of WNV infection from cerebrospinal fluid (CSF). Several whole blood samples and one serum sample underwent further testing. CSF and serum samples were negative for WNV antibody; however, all samples were positive by both RT-PCR assays. Infectious virus was recovered from a blood sample, and its identity was confirmed by using a WNV-specific immunofluorescence assay. The complete WNV genomes determined from CSF and from the virus isolate adapted from cell culture were the same. The results represent the first complete WNV genome sequence obtained directly from human CSF and the first time that infectious WNV has been recovered from a patient with encephalitis in North America.

*West Nile virus* (WNV), an arthropod-borne virus, is a member of the Japanese encephalitis virus serocomplex of the genus *Flavivirus*, family *Flaviviridae* (1), discovered in Uganda in 1937 (2). Although WNV infections are usually mild or asymptomatic, in some instances, a severe and fatal encephalitis is produced, typically in the elderly (3). This virus was first recognized in the Western Hemisphere in an outbreak in New York in 1999 (4). As of October 2, 2002, a total of 2.671 cases of human illness in the United States have been reported to the Centers for Disease Control and Prevention (CDC). Although WNV has been recovered from mosquitoes, birds, and horses, no isolations from humans have been reported in the Western Hemisphere. Thus, all prior data regarding the virus responsible for human illness in North America have come from nucleic acid sequencing of the viral genome in either cerebrospinal fluid (CSF) or brain tissue. We describe the first isolation of WNV from a human case-patient associated with the U.S. outbreak. Because the virus was also directly detected by reverse transcription–polymerase chain reaction (RT-PCR) in specimens from the same patient, we were able to compare the entire genomic sequence of the directly detected virus with that of the cell-culture isolate.

## Case Report

The patient was a 70-year-old woman, who has been diagnosed with intermediate-grade, CD 20-positive, B-cell non-Hodgkin lymphoma, involving a left intraparotid lymph node. Staging workup demonstrated stage 1-A disease. Treatment plans for the patient included three courses of CHOP (cyclophosphamide, hydroxydoxorubicin, vincristine [Oncovin], and predisone) chemotherapy plus rituximab (chimeric CD 20 monoclonal antibody), to be followed by involved field radiation. After the first cycle of chemotherapy in July 2001, neutropenia developed in the patient after both the second and third cycles of chemochemotherapy. The third cycle of chemotherapy was administered on September 11, 2001. Four days later, she was treated with oral levofloxacin for low-grade fever. On day 7 after chemotherapy, she was admitted to the hospital with fever, cough, chills, rhinorrhea, joint aches, decreased appetite, lethargy, and lightheadedness. She had no recollection of any ill contacts or insect bites. She was a resident of southern Nassau County, New York, and did not have a history of travel. Physical examination was within normal limits. The patient was neutropenic; G-CSF was continued, and she was given cefipime and gentamicin. One day after admission, she continued to have fever and experienced mild headaches that responded to acetaminophen. Urine cultures showed penicillin-sensitive enterococci, and blood cultures were negative. Two days after admission, the patient continued to have fever, headaches, and dizziness. A computed tomography (CT) scan of the brain revealed no acute cerebral processes. On the 3rd day after admission, the patient was noted to be confused. Further deterioration of mental status was noted, with incomprehensible speech, but she was able to follow commands. Arterial blood-gas analysis showed an acute respiratory acidosis pattern, and the patient was subsequently intubated and transferred to the intensive care unit. The patient had a hypotensive episode secondary to atrial flutter, which required cardioversion for stabilization. Ceftriaxone and ampicillin were added to the antibiotic coverage, and antifungal treatment was also initiated with lipid complex amphotericin B. After the patient’s hematologic parameters improved, G-CSF was discontinued on day 9 of hospitalization, and the patient continued to be afebrile. Because of the clinical picture of encephalitis and because the patient lived in an area where WNV was endemic, lumbar puncture was performed on day 8, and a CSF specimen was sent to the New York State Department of Health laboratories for comprehensive PCR testing. Renal tubular necrosis developed in the patient, leading to acute renal failure by day 10, with further deterioration of her mental status. Dialysis was initiated on day 16, and antibiotic therapy was discontinued on day 17 as the patient remained afebrile and the neutropenia was resolved. The patient remained unresponsive, staphylococcal septicemia developed, and she died on day 35 of hospitalization. Autopsy showed a small focus of perivascular lymphocyte cuffing in the mamillary bodies of the brain, consistent with viral encephalitis. No evidence of residual lymphoma was found.

## Materials and Methods

The PCR Laboratory associated with the Virology Diagnostic Services, Wadsworth Center, New York State Department of Health, has developed a panel of PCR and RT-PCR assays that allow tests on CSF and brain tissue for a wide range of viruses associated with human central nervous system (CNS) infections. Specifically, the test battery includes herpes simplex viruses (types 1 and 2), varicella-zoster virus, cytomegalovirus, Epstein-Barr virus (*Human herpesvirus 4*), enteroviruses, and the following arboviruses: Eastern equine encephalitis, California serogroup (LaCrosse virus [LACV], Jamestown Canyon, and others), *Powassan virus* (POWV), *St. Louis encephalitis virus* (SLEV), WNV, and Cache Valley virus.

RNA and DNA were simultaneously extracted from 0.25 mL of CSF sample with Trizol LS reagent (Invitrogen, Carlsbad, CA) according to the manufacturer’s instructions. Briefly, RNA was first transcribed into cDNA with random primers (Roche Diagnostics Corp., Indianapolis, IN), and an aliquot of this cDNA (5 μL) was used in PCR reactions with primers for detecting viruses in the test panel. Aliquots of DNA were also examined for the presence of the herpesviruses in the panel. Amplification products were analyzed on a 2% agarose (EM Science, Gibbstown, NJ) gel containing ethidium bromide. Routinely, for any virus for which a band corresponding to a positive result is observed, the band is run into low melting agarose, and sequenced directly on the gel slice without further purification. DyeTerminator sequencing was performed on an Applied Biosystems Model 373A automated sequencer (Foster City, CA). For the TaqMan assay, one-step RT-PCR Ready-Mix Kit (Applied Biosystems) was used. The primers and probe for the quantification of the RNA copy number of WNV used in this study are listed in Table 1.

**Table 1:** Oligonucleotide primers and probe used in the standard RT-PCR and TaqMan assays^a^

Primer	Genome target	Genome position^b^	Sequence (5′–3′)	RT-PCR product size (bp)
CU9093	NS_5_	9097–9120	AGYMGRGCHATHTGGTWYATGTGG	206
CL9279	NS_5_	9302–9283	TTCCAVCCDGCKGTRTCATC	
				
D87F	NS_5_	10034–10051	GCTCCGCTGTCCCTGTGA	70
D156R	NS_5_	10103–10083	CACTCTCCTCCTGCATGGATG	
				
Forwardddd	ENV	1160–1180	TCAGCGATCTCTCCACCAAAG	70
Reverse	ENV	1229–1209	GGGTCAGCACGTTTGTCATTG	
Probe	ENV	1186–1207	TGCCCGACCATGGGAGAAGCTC	

^a^RT-PCR, reverse transcription–polymerase chain reaction. ^b^Genome position according to GenBank accession no. AF196835.

Virus was isolated by using monolayers of Vero cells grown in tubes. Aliquots of 0.01 and 0.05 mL of whole blood were pretreated with antibiotics (penicillin and streptomycin) for 30 min at room temperature (22°C); 1 mL of Eagle's minimal essential medium containing 2% fetal bovine serum was added to the treated blood samples, and the mixtures were used to inoculate the Vero cell monolayers.

After incubation for 24 hr at 37°C, the monolayers were rinsed with phosphate-buffered saline, 2 mL of fresh medium was added, and the culture was subsequently monitored daily for cytopathic effects (CPE). Cell monolayers that showed CPE were harvested. To confirm that the infectious agent was WNV, an aliquot of the supernatant serially diluted (10^-4^–10^-6^) was used to infect fresh Vero cell monolayers. Virus-infected monolayers from the second passage were examined for WNV antigen by immunofluorescence assay (IFA) by using monoclonal antibody H5-46 (provided by CDC, Fort Collins, CO). This monoclonal immunoglobulin (Ig) M antibody is specific for a glycoprotein epitope on WNV.

## Results

### Diagnosis of WNV Infection

**Figure 1:**
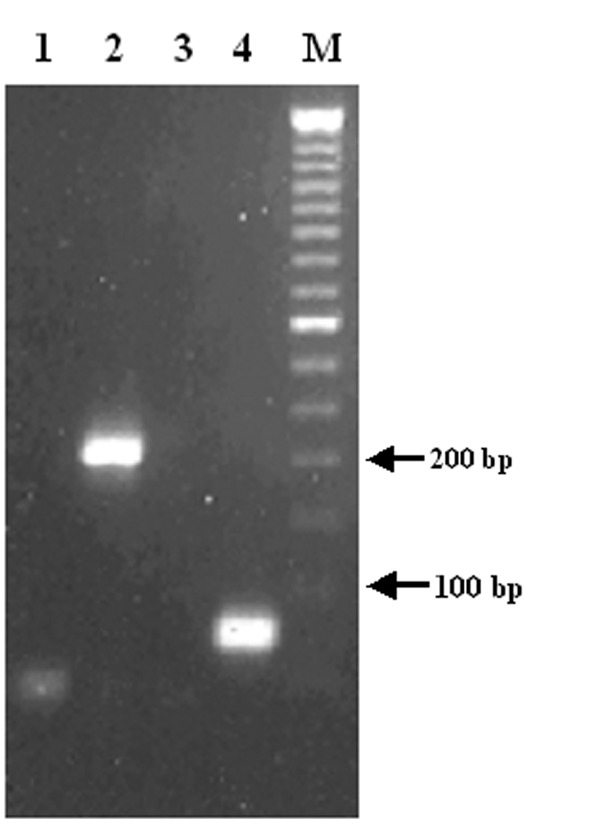
RT-PCR detection of *West Nile virus* RNA in cerebrospinal fluid. Lanes 1 and 3: negative controls; lanes 2 and 4: cerebrospinal fluid; lane M: 50-bp DNA ladder. Primer pairs used: lanes 1 and 2: CU9093/CL9279; lanes 3 and 4: D87F/D156R.

CSF was simultaneously examined for a panel of 11 viruses by RT-PCR/PCR as described in Material and Methods. No amplification product was observed for any other virus in the PCR battery except WNV. Two primer pairs (CU9093/CL9279 and D87F/D156R) in the NS_5_ region (5,6) of the WNV genome were used in the initial screening by standard RT-PCR (Table 1). PCR products of appropriate size for both primer pairs were obtained (Figure 1). RT-PCR was independently repeated on another aliquot of CSF by using four primer pairs located in various genomic regions of WNV. PCR bands with the expected sizes were present in all four reactions (data not shown). The identity of the PCR bands was confirmed by sequence data obtained directly from the PCR amplicon. The diagnosis of WNV infection was based on the detection and sequence of the WNV genome in CSF. Although the serologic test (IgM capture enzyme-linked immunosorbent assay [ELISA]) on the CSF sample was negative for WNV, according to the interpretations set forth by CDC, the detection of viral genome sequence in CSF meets the definition of a confirmed case.

### TaqMan Assays

To follow up this case, three whole-blood samples and one serum sample were examined by both standard RT-PCR (primers in NS_5_ region) and TaqMan (primers in ENV region) assays; the results are summarized in Table 2. The highest RNA copy number, 2.5 x 10^6^ copies/mL, was found in the blood sample that was collected 3 days after the patient’s neurologic symptoms appeared. WNV genome was also detected in a serum sample collected on day 19 after onset of symptoms. Serologic tests (IgM capture ELISA and IgG ELISA) on serum for *Eastern equine encephalitis virus*, LACV, POWV, SLEV, and WNV were all negative.

**Table 2:** Detection of *West Nile virus* in human specimens by TaqMan and standard RT-PCR assays^a^

Specimen	Collection date	RNA (copy/mL)	C_T_	Rn	STD RT-PCR	Serology	Cell cultures
Blood	9/21/2001	2.2 x 10^3^	31.6	0.48	Positive	n.d.	n.d.
Blood	9/24/2001	2.5 x 10^6^	20.4	3.71	Positive	n.d.	Positive
CSF	9/26/2001	1.1 x 10^6^	20.4	3.71	Positive	Negative	Negative
Blood	10/02/2001	5.4 x 10^4^	25.8	2.70	Positive	n.d.	n.d.
Serum	10/10/2001	3.7 x 10^3^	28.5	0.70	Positive	Negative	n.d.

^a^C_T_, threshold cycle number, the cycle number at which fluorescence increases above a fixed threshold value; Rn, normalized fluorescent signal, the fluorescent signal generated by the reporter dye; STD, standard; RT-PCR, reverse transcription–polymerase chain reaction; n.d.: not done.

### Other Clinical Data <H2>

**Figure 2:**
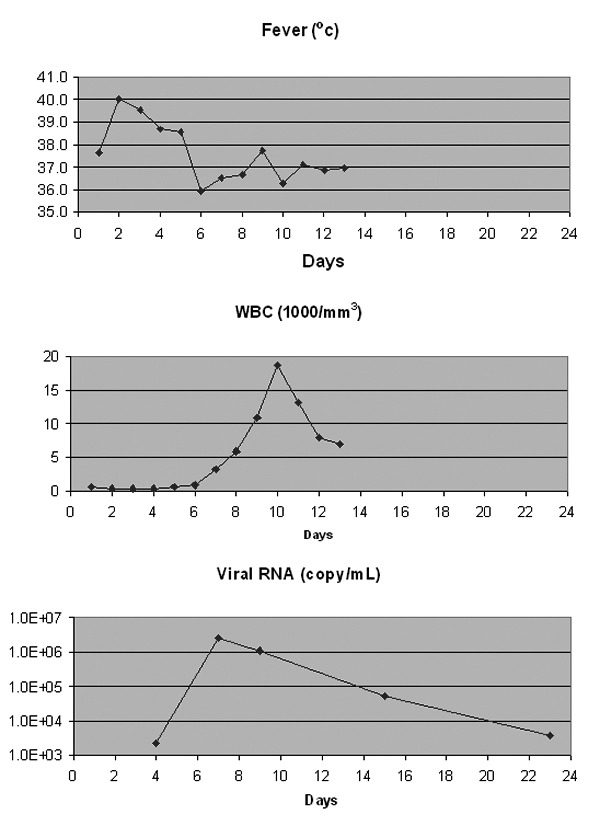
*West Nile virus* copy numbers in clinical samples and clinical indications. WBC, leukocytes. Detailed sample information is listed in Table 2; day 1 is date the patient was hospitalized, 9/18/2001.

Laboratory studies of the CSF indicated the following values: leukocyte (WBC) count 8 mm^3^ with 66% neutrophils, 4% lymphocytes, 4% atypical lymphocytes, and 26% monocytes; erythrocyte count 0; glucose level 76 mg/dL; total protein level 55 mg/dL, and lactate dehydrogenase level 35 IU/L. Figure 2 presents fever curve, WBC curve, and viremia data. The serum immunoglobulins at the time of infection with WNV were IgG 492 mg/dL (normal level [nl]) 700–1,500 mg/dL), IgA 86 mg/dL (nl 65–450 mg/dL), and IgM 80 mg/dL (nl 45–230 mg/dL).

### Virus Isolation

The attempt to isolate WNV was carried out in a biosafety level 3 laboratory not routinely used for arbovirus work and located in a building separate from the facility where PCR testing was conducted. This procedure had the dual advantage of minimizing the possibility that any virus recovered originated from a source other than the human specimen and also ensuring that any isolate obtained would not lead to future spurious PCR results. A blood sample collected on September 24, 2001, and a CSF specimen collected on September 26, 2001, were chosen for recovery of the virus because of the presence of high-copy-number viral RNA. On day 6 postinfection, CPE was observed in the tubes inoculated with 0.01 mL and 0.05 mL of blood, but not in the tube inoculated with 0.1 mL of CSF. WNV was confirmed in the second-passage cell cultures by IFA by using WNV-specific monoclonal antibody H5-46.

### Sequence Analysis

**Figure 3:**
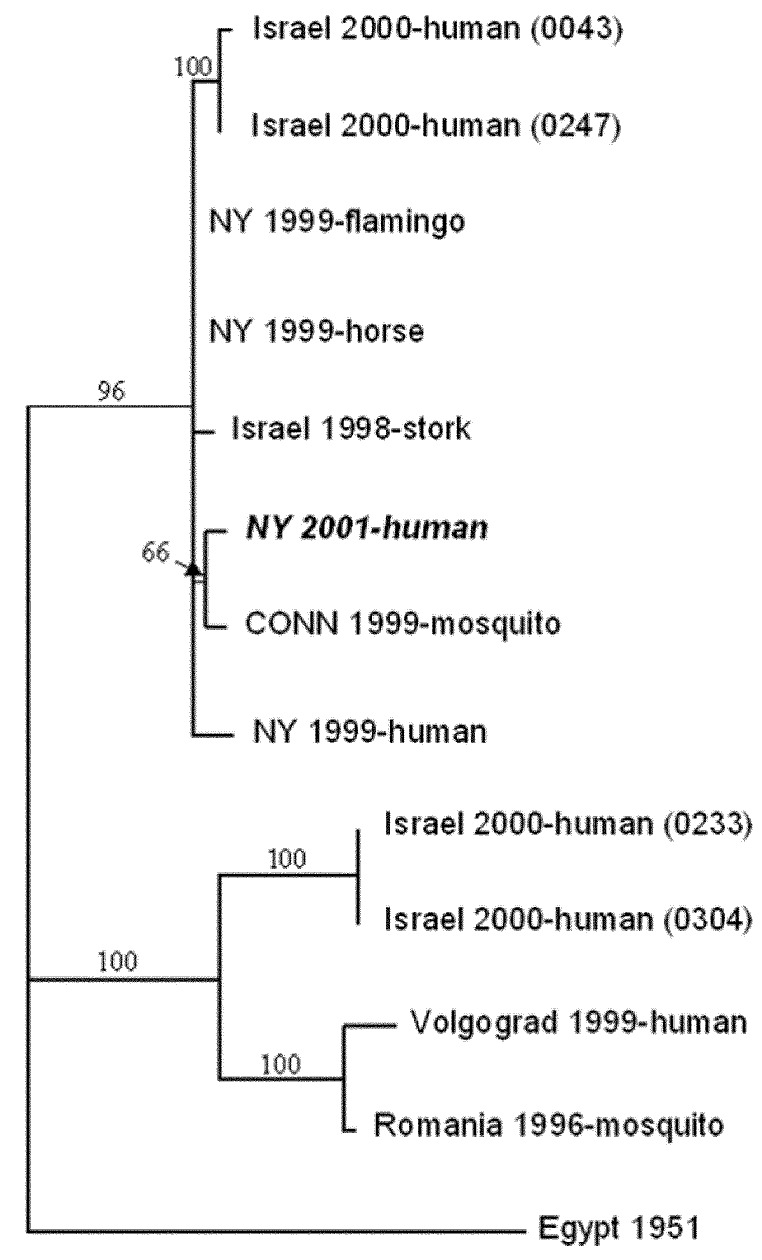
Phylogenetic relationships among *West Nile virus* strains. Sequence data from the present case are shown in italics. The tree is based on the 1,648-bp fragment encoding the preM, M, and part of the 5′-E gene. Numbers at the nodes are bootstrap confidence estimates based on 1,000 replicates.

Since only a very small amount of CSF remained after the diagnostic work-up, we carefully designed a protocol to generate PCR bands with sizes ranging from 500 to 1000 bp. This protocol allowed the complete genome sequence of the virus in the CSF to be determined by sequencing overlapping PCR bands (GenBank accession no. AF533540). We used a similar protocol to obtain the genome sequence of the isolate adapted from cell culture and found that the sequence data from the virus in the CSF and from the WNV isolate were identical. A 1648-bp fragment encoding the PreM, M, and part of the 5′-E gene was used for phylogenetic studies (Figure 3). The analysis showed that the sequence data from this case are similar to the sequence data obtained from human (7) (GenBank accession no. AF202541), horse (8) GenBank accession no. AF260967), and bird (9) (GenBank accession no. AF196835) WNV isolates in New York in 1999.

## Discussion

This case is important for several reasons. It represents the first instance in which WNV was recovered from a person in the United States. It is also the first time that the entire genomic sequence of WNV has been obtained from CSF from a human case-patient. The sequence data from the virus directly detected in the CSF and from the WNV isolate from cell cultures are identical. Hindiyeh et al. (10) reported the isolation of WNV from the blood of viremic patients who were not immunocompromised and who seroconverted later. In contrast**,** the patient in this case was elderly and was undergoing treatment for lymphoma. She was unable to mount an immune response as shown by the fact that the results of serologic tests on both CSF and serum specimens were negative.

To our knowledge, all previous attempts to recover WNV from human patients associated with the North American outbreak have been unsuccessful. The ability to recover an infectious isolate in this report may have been contingent on the fact that the patient was immunologically impaired. She had lymphoma and had been undergoing treatment with CHOP plus rituximab for 2 months at the time the WNV infection developed. The relative role of immunosuppression caused by the lymphoma itself compared with the immunosuppression due to treatment of the lymphoma is unclear. At the time of the WNV infection, the patient’s serum IgG levels were moderately suppressed. This condition was most likely the result of lymphoma**,** because a reduction in immunoglobulins**,** secondary to impaired B-cell function from rituximab**,** usually occurs after 3 months of therapy (11). In a previous randomized study, elderly patients receiving CHOP chemotherapy and rituximab had increased their overall survival and had not experienced an increase in toxic clinical effects compared with effects from CHOP treatment alone (12). However, rituximab used as a single agent has been reported to lead to excessive bacterial and viral infections, including respiratory tract infections and herpes (13). Rituximab is also implicated as a risk factor for unusual viral infections when used as an immunotherapy agent in the peritransplant period of autologous stem cell transplant in non-Hodgkin lymphoma patients (14). Another consideration is the fact that the patient was neutropenic. The relationship between neutrophil function and the severity of WNV infection is unknown. The virus may be cleared by neutrophils**,** and the severity of the viral infection may have been due to the fact that the patient was neutropenic at the time of the acute infection. What lends credence to this hypothesis is the observation that the highest viral titer as determined by PCR coincided with the recovery of the WBC count. Following the resolution of the myelosuppression, the RNA copy number of the WNV in blood samples declined rapidly (from 1.1 x 10^6^ to 5.4 x 10^4^ copies/mL).

In summary**,** this report is the first of WN encephalopathy in an immunocompromised patient undergoing treatment for lymphoma. The patient’s serologic tests remained negative**,** and the diagnosis was made by RT-PCR from both CSF and peripheral blood, and by in vitro cultivation of the virus from blood. WNV infection should therefore be considered in the differential diagnosis of patients with lymphoma who exhibit encephalopathy, even if serologic tests are negative for WNV. Extra care should be taken to prevent patients with lymphoma, especially those undergoing treatment, from being exposed to WNV.

## References

[1] Calisher CH, Karabatsos N, Dalrymple JM, Shope RE, Porterfield JS, Westaway EG, Antigenic relationships between flaviviruses as determined by cross-neutralization tests with polyclonal antisera. J Gen Virol. 1989;70:37–43. 10.1099/0022-1317-70-1-37 2543738

[2] Smithburn KC, Hughes TP, Burke AW, Paul JH. A neurotropic virus isolated from the blood of a native of Uganda. Am J Trop Med Hyg. 1940;20:471–92.

[3] Hayes CG. West Nile fever. In: T.P. Monath, editor. The arboviruses: epidemiology and ecology. Vol. 7. Boca Raton (FL): CRC Press Inc.;1989. p.59–88.

[4] Centers for Disease Control and Prevention. 1999 Outbreak of West Nile-like viral encephalitis—New York. MMWR Morb Mortal Wkly Rep. 1999;48:845–9. 10563521

[5] Briese T, Jia XY, Huang C, Grady LJ, Lipkin WI. Identification of a Kunjin/West Nile-like flavivirus in brains of patients with New York encephalitis. Lancet. 1999;354:1261–2. 10.1016/S0140-6736(99)04576-6 10520637

[6] Briese T, Glass WG, Lipkin WI. Detection of West Nile sequences in cerebrospinal fluid. Lancet. 2000;355:1614–5. 10.1016/S0140-6736(00)02220-0 10821368

[7] Jia XY, Briese T, Jordan I, Rambaut A, Chi HC, Mackenzie JS, Genetic analysis of West Nile New York 1999 encephalitis virus. Lancet. 1999;354:1971–2. 10.1016/S0140-6736(99)05384-2 10622305

[8] Lanciotti RS, Ebel GD, Deubel V, Kerst AJ, Murri S, Meyer R, Complete genome sequences and phylogenetic analysis of West Nile virus strains isolated from the United States, Europe, and the Middle East. Virology. 2002;298:96–105. 10.1006/viro.2002.1449 12093177

[9] Lanciotti RS, Roehrig JT, Deubel V, Smith J, Parker M, Steele K, Origin of the West Nile virus responsible for an outbreak of encephalitis in the northeastern U. S. Science. 1999;1286:2333–7. 10.1126/science.286.5448.2333 10600742

[10] Hindiyeh M, Shulman LM, Mendelson E, Weiss L, Grossman Z, Bin H. Isolation and characterization of West Nile virus from the blood of viremic patients during the 2000 outbreak in Israel. Emerg Infect Dis. 2001;7:748–50. 11585544 10.3201/eid0704.010428 PMC2631769

[11] Mclaughlin P, Grillo-Lopez AJ, Link BK, Levy R, Czuczman MS, Williams ME, Rituximab chimeric anti-CH20 monoclonal antibody therapy for relapsed indolent lymphoma: half of patients respond to a four-dose treatment program. J Clin Oncol. 1998;16:2825–33. 9704735 10.1200/JCO.1998.16.8.2825

[12] Coiffier B, Lepage E, Briere J, Herbrecht R, Tilly H, Bouabdallah R, CHOP chemotherapy plus rituximab compared with CHOP alone in elderly patients with diffuse large-B-cell lymphoma. N Engl J Med. 2002;346:235–42. 10.1056/NEJMoa011795 11807147

[13] Foran JM, Rahatiner AZS, Cunningham D, Popescu RA, Solal-Celigny P, Ghielmini M, European phase-II study of rituximab (chimeric CD20 monoclonal antibody) for patients with newly diagnosed mantle-cell lymphoma and previously treated mantle-cell lymphoma, immunocytoma, and small B-cell lymphocytic lymphoma. J Clin Oncol. 2000;18:317–24. 10637245 10.1200/JCO.2000.18.2.317

[14] Goldberg SL, Pecora AL, Alter RS, Kroll MS, Rowley SD, Waintraub SE, Unusual viral infections (progressive multifocal lukoencephalopathy and cytomegalovirus disease) after high dose chemotherapy with autologous stem cell rescue and peritransplantation rituximab. Blood. 2002;99:1486–8. 10.1182/blood.V99.4.1486 11830505

